# Prevalence of Vitamin D Deficiency among Adult Patients in A Tertiary Care Hospital

**DOI:** 10.31729/jnma.4534

**Published:** 2019-08-31

**Authors:** Chandra Kala Rai, Biju Shrestha, Jyotshna Sapkota, Jay Kumar Das

**Affiliations:** 1 Department of Physiology, Kathmandu Medical College, Duwakot, Bhaktapur, Nepal; 2 Department of Microbiology, Kathmandu Medical College, Duwakot, Bhaktapur, Nepal; 3 Department of Pathology, Kathmandu Medical College and Teaching Hospital, Sinamangal, Kathmandu, Nepal

**Keywords:** *prevalence*, *vitamin D*, *vitamin D deficiency*

## Abstract

**Introduction:**

The deficiency of vitamin D is major public health problem worldwide. It is deficiency of vitamin D level when blood serum which is below 30ng/ml. The deficiency is associated with various musculoskeletal diseases and autoimmune diseases. The early detection of deficiency plays important role to prevent those diseases. The aim of the study is to find the prevalence of vitamin D deficiency among adult population in a tertiary care hospital.

**Methods:**

This descriptive cross-sectional study was conducted in tertiary care hospital, from 1^st^ August 2017 to 31^st^ December 2018 after ethical clearance from institutional review committee with registration number 02082017. Simple random sampling was done. Data was collected and entered in statistical package for social sciences. Point estimate at 95% Confidence Interval was calculated along with frequency and proportion for binary data.

**Results:**

Out of total patients, vitamin D deficiency was found among 283 (73.6%) patients at 95% of CI (68.6-78.6). Out of total female patients, 202 (52.61%) were deficient and out of total male patients, 81 (21.08%) were deficient. The mean age±SD of patients was 41.45±16.016 years.

**Conclusions:**

The prevalence of vitamin D deficiency was high compared to previous studies. Vitamin D deficiency was found to be higher in females than males.

## INTRODUCTION

Vitamin D deficiency is recognized as a major public health problem in the world. Vitamin D maintains calcium homeostasis of bone. Low concentrations of vitamin D lead to alterations in calcium and phosphorous homeostasis and results osteoporosis.^1^ The deficiency is due to inadequate exposure to the sun and lack of vitamin D rich diet. It is associated with risk of autoimmune diseases, hypertension, and infectious diseases.^2^ Inadequate/insufficient vitamin D is <75 nmol/l (30 ng/ mL).^3^

Various studies in vitamin D deficiency showed the rate of prevalence is up to 50-90% in both tropical and sub-tropical population.^4^ Increased deficiency level is the major indicator for metabolic syndrome, cardiovascular disease (CVD), musculoskeletal disorder and osteoporosis.^5^ Early finding of vitamin D deficiency and supplement can decrease the risk of many diseases and mortality rate.^6^

The main objective of this study was to find out prevalence of vitamin D deficiency in adults patient in Kathmandu Medical College and Teaching Hospital.

## METHODS

This is a descriptive cross-sectional study which was performed among OPD patients in Department of Pathology, Kathmandu Medical College and Teaching Hospital, Sinamangal, Kathmandu, Nepal from 1^st^ August 2017 to 31^st^ December 2018.The ethical approval was received from Institutional Review Committee of Kathmandu Medical College and Teaching Hospital (Ref:02082017). Study population of this study are all the OPD patients of age group 15 to 75 years were included. Patients above 75 years and below 15 years, chronic disease and malignancy were excluded.

Level of vitamin D is tested by quantitative method, chemiluminescent immunoassay (CLIA) methods. Blood sample (5 mL) was taken in gel tube for each subject and serum was separated by centrifuge to estimate vitamin D [25 (OH)D]. Vitamin D estimation was performed in the pathology lab by chemiluminescent immunoassay (CLIA). The value less than 30 ng/ml was considered as deficient and above it was considered non-deficient.

Simple random sampling was done and sample size was calculated using the following formula,

n = Z^2^ × pq/e^2^

Where,

n = sample size
Z= 1.96 for 95% Confidence interval
p = prevalence, 50%
q = 1-p
e= margin of error= 5%

Based on the above formula, the minimum samples size was 384 and further subgroup analysis was done on the basis of age and gender.

Selection and information bias has been minimized as possible. Data was analyzed by using Statistical Package of Social Science (SPSS) software version 16, point estimate at 95% CI was calculated along with frequency and proportion for binary data.

## RESULTS

**Figure 1:**
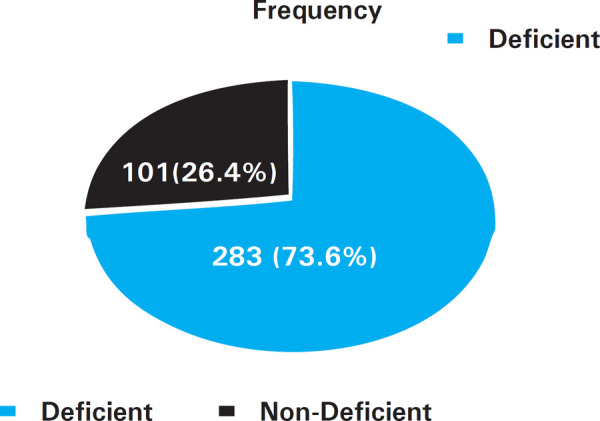
Prevalence of Vitamin D deficiency among total patients.

Out of total 384 patients, vitamin D deficiency was found among 283 (73.6%) patients at 95% CI (68.678.6) (Figure 1). Out of total female patients, 202 (52.61%) were deficient and out of total male patients 81 (21.08%) were deficient (Table 1). Mean age±SD of patients age from 15-75 years, was estimated to be 40.45±16.016 years. Total of 384 patients were included [Male: 108 (28.09%) and Female: 276 (71.89%)] from OPD of Kathmandu Medical College and Teaching Hospital, Sinamangal.

**Table 1:** Prevalence of Vitamin D deficiency in different gender.

Gender	Vitamin Deficient n (%)	Vitamin Non-Deficient n (%)	Total n (%)
Female	202 (52.61)	74 (19.28)	276 (71.89)
Male	81 (21.08)	27 (7.01)	108 (28.09)
Total	283 (73.3)	101 (26.3)	384 (100)

## DISCUSSION

In our findings, the prevalence of vitamin D deficiency were found to be similar by Kurt A. Kennel 73%.^7^ A study done by Forrest KY showed prevalence of 41.65% in US adults. Maximum 82.1% deficiency was seen black population.^8^ A study done in Beijing by Ning Z. et.al showed the prevalence of deficiency by 87.1%.^8^ A study in France showed 92.3% of participants were deficient of vitamin D.^9^

Similar to this study, a study done in Beijing showed the higher rate of prevalence in female 89.0% and male was 84.9%.^10^ A study of Saudi Arabia showed prevalence of male deficient was 87.8%.^11^ A study of Northern Vietnam showed the prevalence of vitamin D deficiency in women was 30%, almost two-fold higher than in men (16%).^12^

The limitation of the study was carried out on only one hospital and thus cannot be generalized. The research would be more effective if the personal history of sun exposure and dietary habit were included.

## CONCLUSIONS

Prevalence of Vitamin D deficiency is higher compared to previous studies. The prevalence of Vitamin D deficiency is higher in females than males. If the deficiency can be

## Conflict of Interest:

**None.**
